# Northward shift of the agricultural climate zone under 21^st^-century global climate change

**DOI:** 10.1038/s41598-018-26321-8

**Published:** 2018-05-21

**Authors:** Myron King, Daniel Altdorff, Pengfei Li, Lakshman Galagedara, Joseph Holden, Adrian Unc

**Affiliations:** 1 Environmental Policy Institute, School of Science and the Environment, Memorial University of Newfoundland, Corner Brook, NL A2H 4G5 Canada; 2 School of Science and the Environment, Memorial University of Newfoundland, Corner Brook, NL A2H 4G5 Canada; 3 College of Geomatics, Xi’an University of Science and Technology, Xi’an, 710054 China; 4 water@leeds, School of Geography, University of Leeds, Leeds, LS2 9JT UK

## Abstract

As agricultural regions are threatened by climate change, warming of high latitude regions and increasing food demands may lead to northward expansion of global agriculture. While socio-economic demands and edaphic conditions may govern the expansion, climate is a key limiting factor. Extant literature on future crop projections considers established agricultural regions and is mainly temperature based. We employed growing degree days (GDD), as the physiological link between temperature and crop growth, to assess the global northward shift of agricultural climate zones under 21^st^-century climate change. Using *ClimGen* scenarios for seven global climate models (GCMs), based on greenhouse gas (GHG) emissions and transient GHGs, we delineated the future extent of GDD areas, feasible for small cereals, and assessed the projected changes in rainfall and potential evapotranspiration. By 2099, roughly 76% (55% to 89%) of the boreal region might reach crop feasible GDD conditions, compared to the current 32%. The leading edge of the feasible GDD will shift northwards up to 1200 km by 2099 while the altitudinal shift remains marginal. However, most of the newly gained areas are associated with highly seasonal and monthly variations in climatic water balances, a critical component of any future land-use and management decisions.

## Introduction

A projected consequence of climate change is the decrease of farmland and crop production^1–3^ in established agricultural regions due to more irregular and extreme weather throughout the cropping season^4,5^. Climate change is highly likely to result in an elevated temperature, which is above the stress thresholds for many crops, especially at night^6,7^. However, northward shift of warmer climate^8,9^ might create an opportunity for a re-evaluation of the currently unexploited areas in the boreal region as to their suitability for agriculture^10,11^, regions where arable agriculture is currently not considered feasible^12^. Although a gain of agricultural areas in the northern region has been proposed^10,11^, many land-use projections are confounded by high uncertainties resulting from different biophysical and socio-economic assumptions and modelling approaches, with most uncertainties associated with less surveyed marginal regions, such as boreal regions^13^. In previous studies, the gain of agronomically feasible areas was projected based on assumptions of temporally stable edaphic conditions^10^ and by employing a single climate model, thus not accounting for kinetic and model-related uncertainties. Crop suitability modelling largely depends on a range of highly variable edaphic parameters that are in turn affected by interactions with parameters of a changing climate and management changes^3,14^. Hence, understanding of the areas that might reach climatic feasibility for future agronomic use ought to be the first step for any consideration of a northward extension of agriculture. The projection of the future extent of climatically feasible areas requires suitable understanding of plant available heat units and temperature ranges in relation to crop phenology and growth, both commonly integrated and described by growing degree days (GDD) intervals^15,16^. GDD values are calculated by summing the daily degrees above a reference temperature (T_base_) within the frost-free period^17^. A common T_base_ for colder regions is 5 °C (GDD_5_)^18^, but it varies with crop types. Here we used the GDD_5_ ≥ 1200 (°C) threshold to predict future climatic suitability for crops in boreal regions. The GDD_5_ values were derived from the temperature outputs of seven global climate models (GCMs). Areas with GDD_5_ ≥ 1200 (°C) are considered feasible for small cereal crops, such as barley and oat, and used to describe the minimal climatic requirements for agriculture^9,16,18–21^. In addition, the interaction between the GDD shift and photoperiod needs to be acknowledged^21,22^. Crops are sensitive, neutral or insensitive to photoperiod, with differences that may be dependent upon phenological stages^15,19^. Information about future precipitation patterns and potential evapotranspiration (PET)^23^ during the growing seasons also informs decisions on the feasibility of agronomic activities^24^. Here we aimed to assess the spatial and temporal nature of the expansion of agriculturally feasible climatic conditions in the boreal zone by the end of the 21^st^-century. Thus, we offer a baseline for the identification of regions that might warrant in-depth, structured agronomic research, that also addresses comprehensively crop, edaphic and economic constraints.

## Results

**Figure 1:**
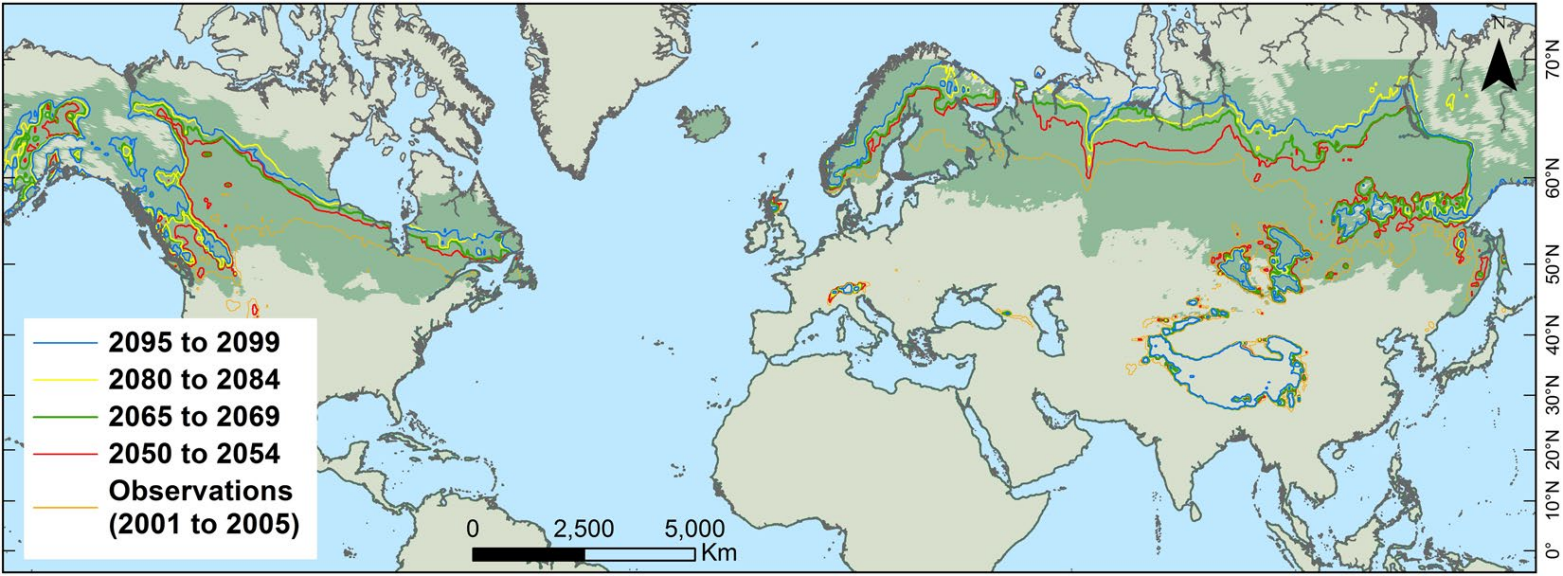
Projected advances of the GDD_5_≥1200 boundary. The green area describes the current extent of the boreal region^66,67^. GDD_5_ values were estimated by averaging the projections of seven CO_2_ emission based GCMs (Extended Data Table 1). Each time step describes a five-year average of all estimates by the seven GCMs. The variability in projection among models is presented in Fig. 4 and Table 1. Map created using ArcGIS Desktop v. 10.4.1^65^.

Projections, which were calculated through averaging the output^25^ of seven GCMs with equal weighting, consistently suggested that GDD_5_ ≥ 1200 areas will expand northward to eventually cover an average of three-quarters of the boreal regions by 2099 (Fig. 1, Table 1). Although temporally and spatially non-linear, the trend is consistent across longitudes. The leading edges of GDD_5_ ≥ 1200 areas in the eastern part of North America, northwest Russia, Finland, and western Asia were found to shift northward by 400 to 600 km by 2099 (Extended Data Fig. 3). A greater linear northward shift was projected for the western parts of North America, with >850 km in places by 2069, and as much as 932 km (Alberta, Canada) by 2099. For large areas of East Asia, a northward shift of up to 1000 km was projected by 2055, reaching nearly 1200 km by 2099 for eastern Siberia (Russian Federation). Notably, the GDD_5_ ≥ 1200 regions along prominent mountain ranges (outside and inside the boreal regions), such as in the Himalayas, the Urals, and the Rockies, remained comparatively stable (Fig. 1, Extended Data Fig. 3).

**Table 1:** Current and projected expansions of GDD_5_ ≥ 1200 areas in the boreal regions of selected countries.

Country	Total land area	Boreal region			Current and projected distribution of the GDD_5_ ≥ 1200 area across the boreal region											
					Current		2050–2054		2065–2069		2080–2084		2095–2099		2005–2099	
		Boreal area^a^	% Boreal		Area^b^	% of boreal	Area	% of boreal (mean [min-max])	Area	% of boreal (mean [min-max])	Area	% of boreal (mean [min-max])	Area	% of boreal (mean [min-max])	Total area gain	% increase above current (mean [min-max])
			of Global	of country												
Russian Federation	16.88	11.53	54.6	68.3	3.94	34.2	7.05	61 [44–70]	7.97	69 [48–78]	8.74	76 [54–86]	9.06	79 [56–87]	5.12	129 [65–155]
Canada	9.96	6.53	30.4	38.7	1.72	26.3	3.62	55 [43–67]	4.07	62 [48–80]	4.50	69 [50–88]	4.79	73 [52–93]	3.07	178 [97–255]
United States	9.47	1.24	5.8	7.4	0.32	26	0.45	36 [31–44]	0.63	51 [34–65]	0.74	59 [36–73]	0.90	72 [44–85]	0.57	177 [68–226]
Mongolia	1.56	0.56	2.6	3.3	0.18	32.9	0.30	53 [40–62]	0.34	60 [42–73]	0.39	70 [50–85]	0.41	734 [50–90]	0.23	125 [51–172]
China	9.41	0.49	2.3	2.9	0.35	71.8	0.47	96 [88–97]	0.47	97 [93–98]	0.48	98 [96–99]	0.48	99 [96–100]	0.13	37 [34–39]
Finland	0.34	0.33	1.5	1.9	0.17	51.2	0.27	83 [70–94]	0.28	86 [72–97]	0.31	94 [79–98]	0.31	95 [80–99]	0.14	84 [55–92]
Sweden	0.45	0.31	1.4	1.8	0.03	8.2	0.13	41 [19–64]	0.16	50 [20–77]	0.20	63 [40–88]	0.20	64 [39–92]	0.17	687 [374–1027]
Norway	0.33	0.28	1.3	1.7	0.03	9.7	0.06	20 [12–31]	0.08	27 [14–52]	0.10	36 [18–71]	0.12	41 [20–78]	0.09	326 [107–704]
Kazakhstan	2.72	0.12	0.6	0.7	0.10	82.8	0.12	92 [88–95]	0.12	96 [92–98]	0.12	97 [93–98]	0.12	98 [95–99]	0.02	18 [15–20]
Kyrgyzstan	0.20	0.02	0.1	0.1	0.01	38.1	0.02	91 [63–98]	0.02	96 [74–100]	0.02	100 [90–100]	0.02	100 [90–100]	0.01	162 [138–163]
Global^c^	—	**21.51**	**100**	—	**6.85**	**32**	**12.47**	**58** [**43–68]**	**14.14**	**66** [**48–78]**	**15.57**	**73** [**55–89]**	**16.40**	**76** [**55–89]**	**9.55**	**140** [**72–180]**

^a,b^Areas in units of 10^6^ km^2^.

^c^Small areas of the boreal regions in other regions are sufficiently small to be masked within the rounding error.

**Figure 2:**
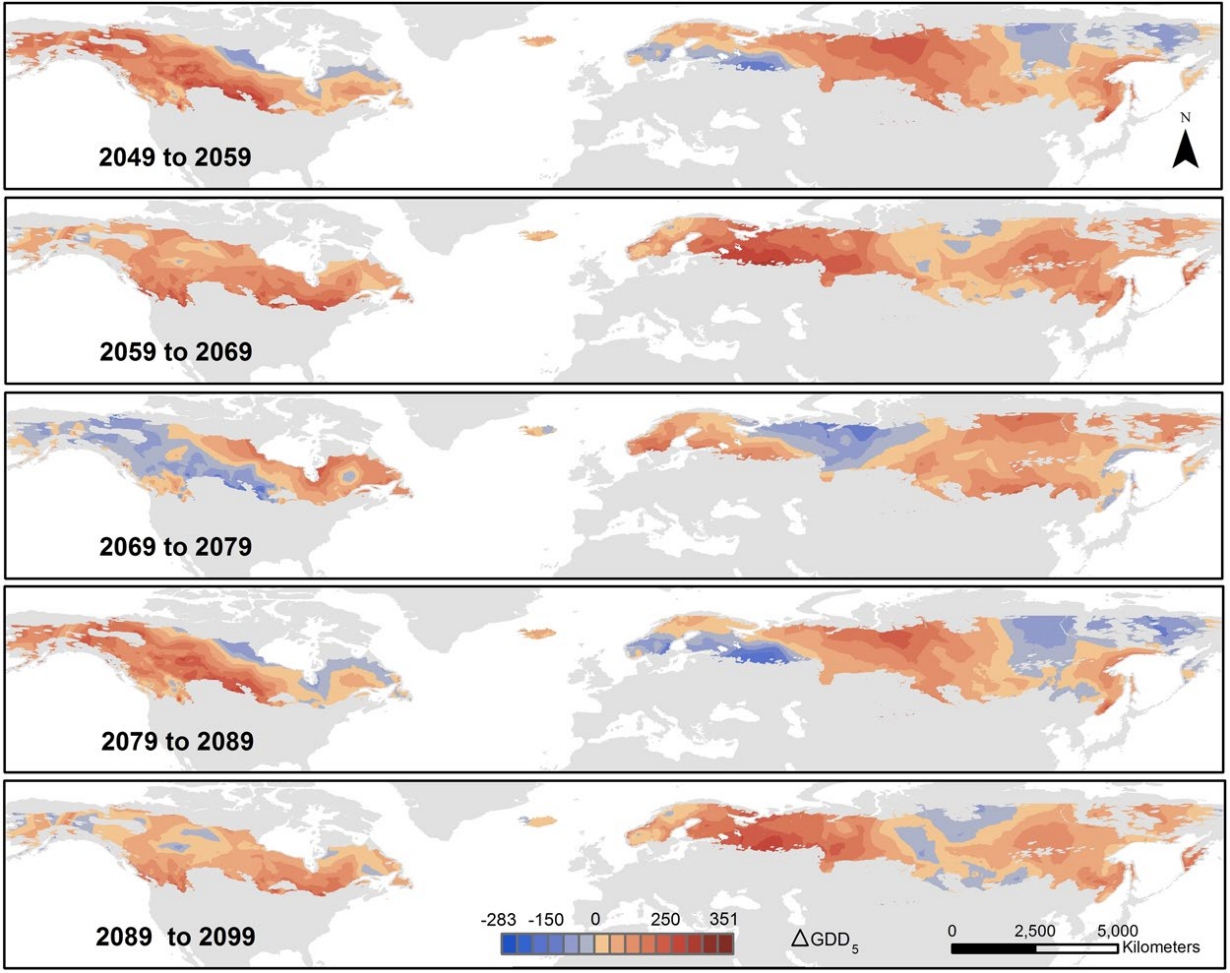
Spatial and temporal variability of GDD_5_ changes within the boreal region as described by selected 10-year period projections. Each map displays the difference between the respective five-year period average values (e.g. 2049 stands for the period 2045–2049). The values were obtained by averaging the emission CO_2_ based projections for the seven GCMs. More detailed steps can be found in the Extended Data (Fig. 7). Map created using ArcGIS Desktop v. 10.4.1^65^.

The rate of the projected expansion varied spatially, temporally and non-linearly as a result of natural climate variability, such as that driven by solar cycles^26^. Comparison of GDD_5_ ≥ 1200 projections for 10-year intervals revealed distinct, alternating spatial patterns with absolute changes ranging from −283 °C to +460 °C (Fig. 2 and Extended Data Fig. 7). With the exception of northern Scandinavia, where the GDD_5_ ≥ 1200 area increases consistently over time, other boreal regions will experience alternating increases and decreases. For example, for the 2049 to 2059 interval, the greatest GDD_5_ ≥ 1200 areal increase is projected for Alaska, the Canadian Northwest Territories, central Canada, and western Siberia, a trend similar to the 2079 to 2089 interval, but more accelerated. For the 2059 to 2069 interval, the GDD_5_ ≥ 1200 areas increase across the entire boreal region, except for parts of central Siberia; the greatest GDD_5_ ≥ 1200 areal increase is forecasted for western Russia and northern United States, a trend similar to the 2089 to 2099 interval. The projections for the 2069 to 2079 interval diverged, with decreased GDD_5_ ≥ 1200 areas expected for large parts of Alaska, central Canada and central Russia.

For certain countries, the GDD_5_ ≥ 1200 areas may rapidly occupy a large proportion of their boreal regions (Table 1). For example, by 2055 the GDD_5_ ≥ 1200 area within the boreal region was found to increase in Kyrgyzstan from the current 38% to 91%, in Sweden from 8% to 41%, and in Finland from 51% to 83%. The projected proportional gain was particularly high for the Scandinavian countries. By the end of the 21^st^-century, the area with GDD_5_ ≥ 1200 was demonstrated to expand by approximately 687% in Sweden (374% to 1027%), 326% in Norway (107% to 704%), and 178% in Canada (97% to 255%). Projections for these countries, however, also had the greatest uncertainties, in part due to the dominantly cross-latitudinal orientation of their territories. The largest absolute increases in the GDD_5_ ≥ 1200 area by the end of the 21^st^-century emerged in the Russian Federation (5.12·10^6^ km^2^), Canada (3.07·10^6^ km^2^) and the USA, mainly in Alaska (0.57·10^6^ km^2^). The other boreal countries were projected to experience a total absolute increase of <0.8·10^6^ km^2^, but which is still relevant for regional land-use planning. Overall, the GDD_5_ ≥ 1200 areas of boreal regions may expand by the end of the 21^st^-century by about 140% (72% to 180%), which equals an area of 9.55·10^6^ km^2^. For Iceland, which covers 0.10·10^6^ km^2^, or 0.5% of the global boreal area, the simulations suggested that it would not be affected; only one of the seven models, ukmo_hadcm3, suggested an expansion of the GDD_5_ ≥ 1200 area, which would cover 15% of Iceland’s boreal area by the end of the 21^st^-century; this is 0.09% of the total gain within the global boreal region.

**Figure 3:**
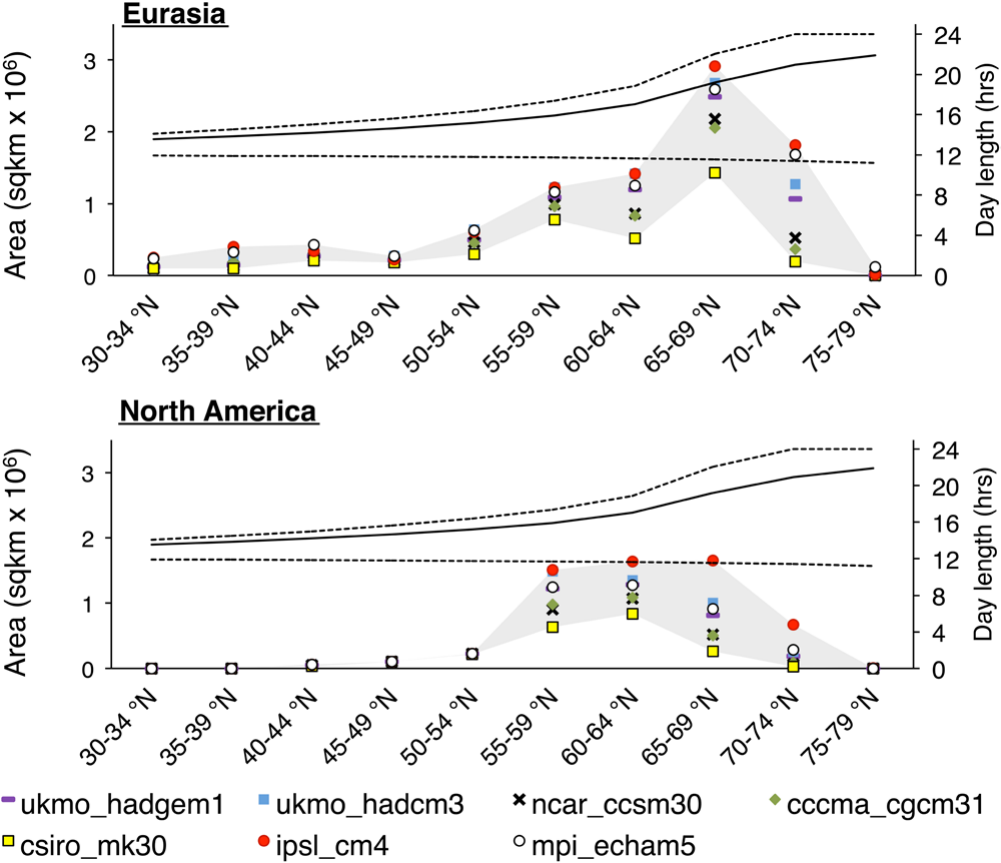
Photoperiods and changes in the GDD_5_ ≥ 1200 area between current conditions and the average projections for 2095–2099. The gray region describes the inter-model variability for the projections calculated by the seven GCMs. The dotted lines represent the maximum and minimum day length, and the continuous line indicates the average day length for May through September at the different latitudes.

**Figure 4:**
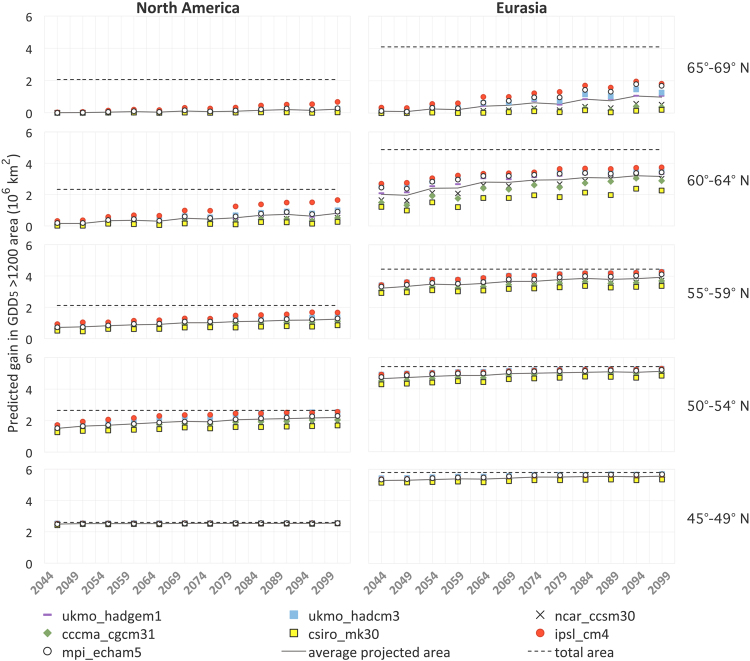
Expansions of the GDD_5_ ≥ 1200 areas as projected by the seven GCMs; graphs include both boreal and non-boreal zones, as distributed across latitudes. All models consistently showed expansion of GDD_5_ ≥ 1200 areas up to the end of the 21^st^ century. Map created using ArcGIS Desktop v. 10.4.1^65^.

Importantly, most projected increases occur in regions that have a different photoperiod profile from current agricultural regions (Fig. 3 and Extended Data Fig. 11). In Eurasia, the GDD_5_ ≥ 1200 area increase within the 65° to 69°N region is projected to be almost twice as large as the new areas projected for all other latitudes. Currently, only 11.6% of the Eurasian and 0.2% of the North American boreal regions above the 54°N parallel have a GDD_5_ above 1200 (Fig. 4). Of the total expansion of GDD_5_ ≥ 1200 area, increases from 57.6% [mpi_echam5] to 71.7% [csiro-mk30] for Eurasia, and from 39.5% [mpi_echam5] to 52.1% [csiro-mk30] for North America were projected for the 55° to 69°N region. However, the largest model deviations were estimated for the highest latitudes (i.e. Fig. 4, Extended Data Fig. 3) as already suggested by the prediction ranges for Scandinavian countries. For example, from 5.2% to 20.8% (mpi_echam5 or csiro-mk30, respectively) of the total Eurasian expansion and from 3.3% to 7.4% (mpi_echam5 or ipsl_cm4, respectively) of the North American expansion is projected for very high latitude regions, above 70°N. The boreal regions currently extend further south in Eurasia than that in North America, and consequently, the predicted temperature shift will result in large areas of GDD_5_ ≥ 1200 in Eurasia within the 30 to 54°N region (from 17.6% [ukmo_hadgem1] to 24.6% [ccma_cgcm31]). In North America, given the current more northerly distribution of GDD_5_, only about 5.3% to 9.4% (ipsl_cm4 and csiro_cm4, respectively) of the total expansion for the GDD_5_ ≥ 1200 is projected to occur in the boreal regions below 54°N.

**Figure 5:**
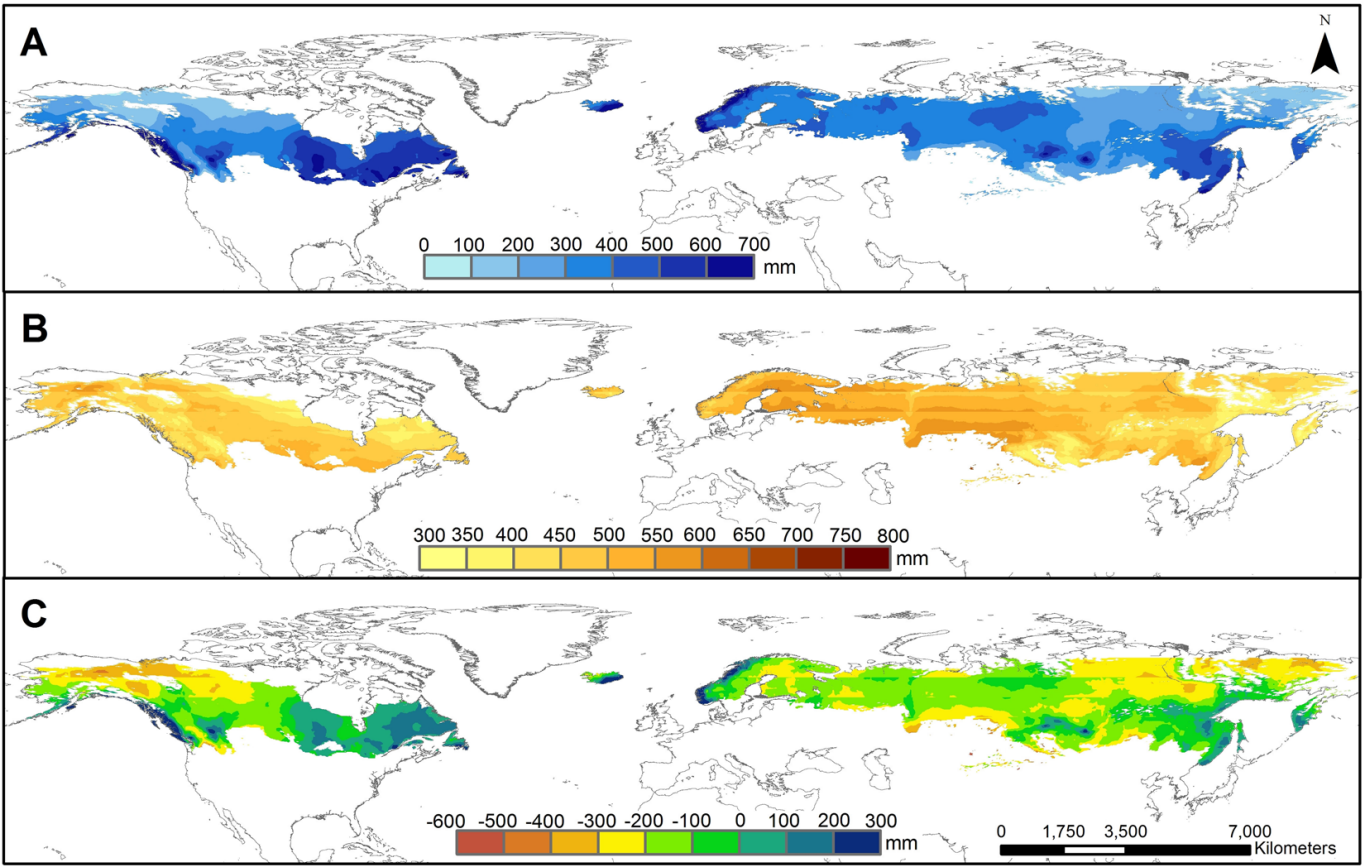
Observed precipitation (**A**) potential evapotranspiration-(PET) (**B**) and climatic water balance (**C**) for May to October of the reference period (2001–2005). Map created using ArcGIS Desktop v. 10.4.1^65^.

**Figure 6:**
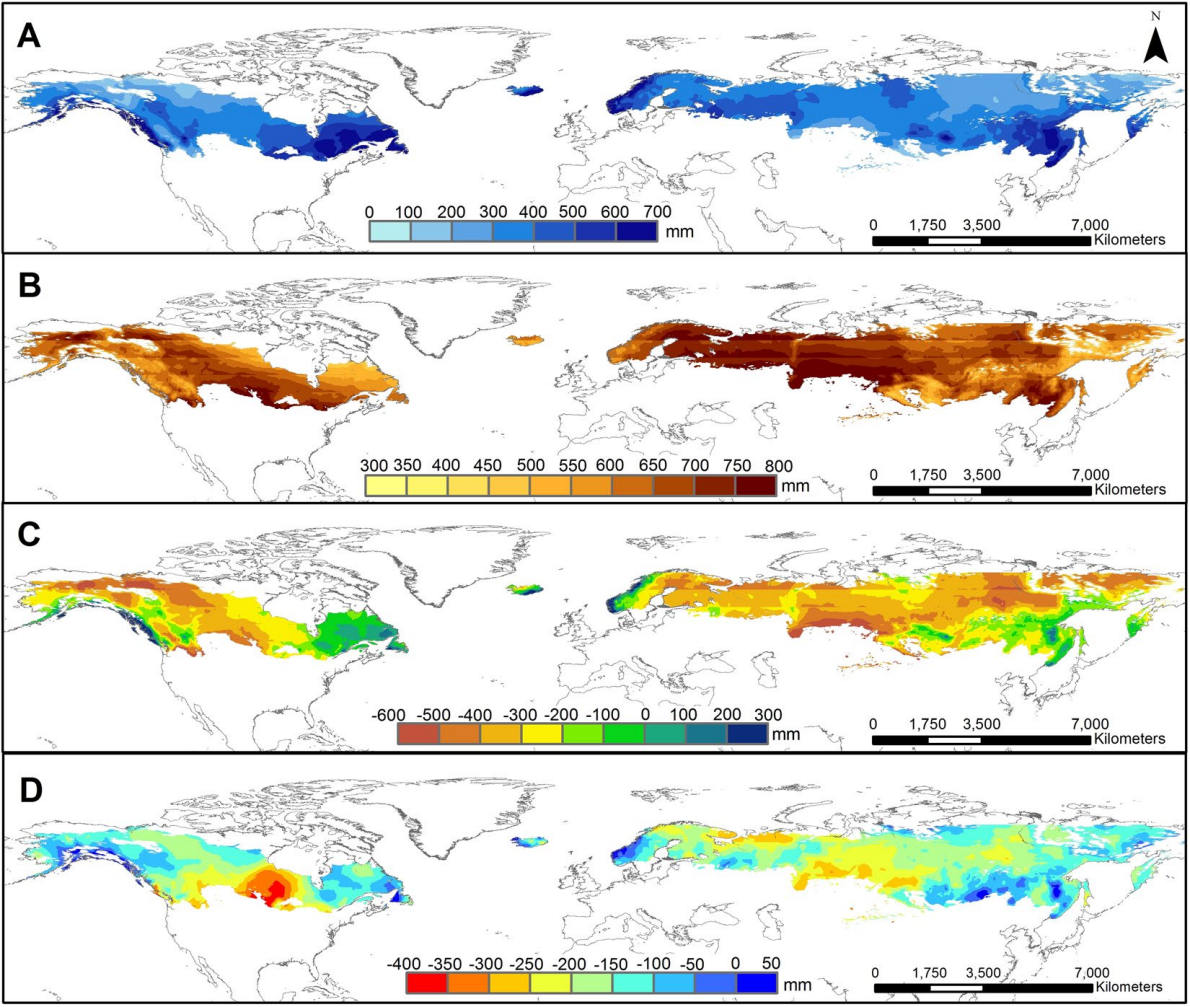
Projected precipitation (**A**) potential evapotranspiration (PET) (**B**) and climatic water balance (**C**) for May to October of 2099. The differences between the projected climatic water balance for 2099 and the observed (2001–2005) are given in (**D**). Stripes in map A and B are an artefact due to the discrete 5° latitude intervals employed in the estimation of PET. Map created using ArcGIS Desktop v. 10.4.1^65^.

A comparison of the current and projected growing season precipitation (Figs 5A and 6A) showed that the spatial pattern remained relatively stable, with future precipitation being slightly higher. Contrary to the relatively stable future precipitation pattern, the climate change associated temperature increase leads to much higher PET across the boreal regions, mostly doubling current PET values (Figs 5B and 6B). This will result in more complex, regionalized climatic water surplus and deficit conditions (Figs 5C and 6C). The highest deficits, resulting from low precipitation and larger PET values, were projected for the central continental regions both of North America and Eurasia. Approximately 60% of the newly gained GDD_5_ ≥ 1200 area in the Eurasian boreal region would experience climatic water deficits during the growing season from May to October (i.e. >300 mm deficit; Fig. 6C and Extended Data Table 3) by the end of the 21^st^-century. A similar scenario was projected for the North American boreal region, where 40% of the GDD_5_ ≥ 1200 area might suffer >300 mm climatic water deficits. Changes in the climatic water balances are expected to be particularly high for large parts of Ontario and Manitoba (Canada) (Fig. 6D). Our detailed spatial and temporal projections, however, indicate that most of the deficits are likely occur for short periods in the summer only and might not meet the recently proposed aridification threshold of <0.65^23^ (Extended Data Table 3 and Figs 8 and 9). In contrast, boreal regions around the Pacific and Atlantic rims were found to receive increasingly more precipitation over the growing season (Fig. 6, Extended Data Figs 8 and 9 and Tables 3 and 4). Especially high regional variability in precipitation during the growing season is projected among the Scandinavian countries (Extended Data Table 4 and Fig. 12). While for the newly gained GDD_5_ ≥ 1200 areas in Norway a surplus of climatic water balance is projected, Sweden, and Finland will likely have deficit of climatic water balance during the growing season. The fall months (September and October), in particular, were found to be associated with climatic water surpluses (Extended Data Fig. 10), even for some regions where an overall climatic water deficit was projected for the growing season.

## Discussion

All simulations consistently showed a geographical upward shift of GDD_5_ ≥ 1200 areas, despite inter-model differences. The projections were particularly different at the northernmost latitudes (Fig. 4 and Extended Data Fig. 2). These differences can be explained by the following: (i) the models were developed based on distinct assumptions regarding ocean currents, interactions between ocean surface and the atmosphere at the boundary layer, and the role and mixing of melting ice, among others^27^ and (ii) the focus of the models is different. For example, the ipsl_cm4 (France) model projected the greatest GDD_5_ changes, for every time step and latitude, whereas csiro_mk30 (Australia) projected the smallest changes. In this case, ipsl_cm4 was calibrated with a focus on the North Atlantic and tropical currents in the northern hemisphere^28^, while the Australian csiro_mk30^29^ was developed and calibrated with a stronger oceanic focus. However, despite inter-model differences in total areas, all trends were uniformly upwards, justifying the use of an inter-model averaging approach^30^. Moreover, the inter-model error, as described by the coefficient of variance, decreased over time (Extended Data Fig. 2).

We found that the projected GDD_5_ ≥ 1200 areas in the boreal zone trebled by the end of the 21^st^-century. While the upward shift of alpine plant species as a consequence of climate change is well documented^31–33^, our results indicated that latitudinal shifts, rather than altitudinal shifts, dominates the projected global expansion of GDD_5_ areas (Fig. 1). The magnitude and progress of the projected northward shift was particularly great for higher latitudes (>50° N), consistent among models (Fig. 3). For some boreal countries (e.g. Finland, Sweden, Kyrgyzstan) the potential for agricultural expansion based on GDD_5_ ≥ 1200 could be transformational to the local land use if this potential is acted upon. However, the temporal variation in the expansion may complicate short-term agricultural development and management planning and practices; inner-continental regions are more likely to undergo a non-linear change in GDD_5_ ≥ 1200 areal expansion, with regular cooling and warming cycles. Such patterns suggest the importance of both, short-term and long-term planning and thus the need for locally relevant approaches to any proposal for an expansion in agricultural land.

Our projections suggest that climatic water availability has strong spatial trends that must be considered when defining agricultural feasibility within the projected GDD_5_ ≥ 1200 areas. Current literature indicates that, on an annualised basis, high latitudes are predicted to receive more precipitation, but of highly variable intensities and non-uniformly distributed^5,34,35^. However, annualised predictions are not adequate for crop management decisions, which are based on seasonal variability in water availability. Our description of the variability of climatic water balances within the growing season indicated that an increase in annual total precipitation does not necessarily mean more water available to crops. Critically, our projections show only marginal gains in precipitation during the growing season, while the projected increase in PET will affect the feasibility of rain-fed cropping for large parts of the newly gained GDD_5_ ≥ 1200 areas. Furthermore, dry spring and summer seasons (planting and vegetative period) and wet falls (harvesting period) might occur (Extended Data, Figs 8 and 9), especially for the inner continental regions, where most of the favourable GDD_5_ expansion is projected. The seasonal climatic water deficits for most of the boreal regions were projected to be between 100 and 600 mm. In comparison, current irrigation needs for yield maximization in northern Europe vary between 0 and 250 mm, with most regions requiring at least some supplemental irrigation^36^. It is likely, that a combination of winter water storage to feed summer irrigation and the introduction and further development of drought-adapted varieties^36,37^ may be needed to support arable expansion in many of the new GDD_5_ ≥ 1200 areas in the boreal zone to 2099. In contrast, boreal regions around the Pacific and Atlantic rims, such as Norway, Atlantic and Pacific Canada, are expected to receive more precipitation and will have larger climatic water surpluses during the growing season, despite an increase in PET. This could be a favourable scenario but may also require substantial investment in agricultural erosion and drainage control measures.

The projections further showed that the shift in the GDD_5_ ≥ 1200 areas occurs unequally across photoperiod profiles and whereas the largest expansion is projected for regions above 55°N some expansion might even occur for areas above 70°N (Fig. 3 and Extended Data Fig. 11). Long daylight hours may offer suitable conditions for cereal crops^38^, but for other crops locally adapted cultivars may have to be evaluated^39^. Any northward shifts of agriculture must therefore consider the need for locally adapted crops^38^ capable of balancing production of total biomass and grain yields facing potential water stress and distinct photoperiods - an area of plant breeding not yet extensively developed^39^.

Boreal land-use change is, of course, restricted by a range of confounding factors. For example, in Canada only about 8.5–9.0% of the current GDD_5_ ≥ 1200 area is used for agriculture, nearly exclusively in the boreal plains of western Canada^40^, of which less than 30% is used for tilled agriculture such as cereals, pulses, or oil crops, with most else used as pasture, for hay or left fallow. The quality of the boreal soils is considered to be the critical limiting factor^40^. While considered as unfavourable for agricultural use boreal soils accumulate and store massive amounts of organic carbon^41–44^. Boreal regions also contain a broad range of unique ecological habitats and species, from microbial communities to vertebrates, subject to complex and fragile interactions^45,46^. It is well documented, that boreal land conversion is associated with negative and often irreversible effects on environmental parameters (soil carbon balances, GHG emissions, and loss of biodiversity)^5,40,42^, and that agriculture has the greatest impact^47^. Hence, any proposed arable conversion of boreal soils should be first subject to a major assessment of potential short and long-term consequences^45–48^, in particular considering global carbon commitments and efforts to halt deforestation and habitat loss proposed by the United Nations^49^ and FAO^50^. There is also a need to monitor the soil functional changes in response to climate change and agricultural conversion^48^ since hydrological processes are coupled with greenhouse gas production and transport. For example, large organic soils regions around the Hudson Bay and James Bay lowlands in Central Canada, Alaska, and in areas of Central Siberia and Northern Europe^51^, are projected to undergo summer water deficits (Fig. 6) which is known to accelerate soil carbon losses^5,41^, even without any conversion to agricultural land use.

Global and local social^52^ and political circumstances^11,52^, will also be important factors governing any changes to boreal agriculture. A balance between global food security requirements in a changing climate^3,53^ and boreal agricultural expansion driven by local food security concerns will likely govern the decisions to expand agriculture towards higher latitudes^11^. As an example, the Government of Newfoundland and Labrador in Canada is currently pursuing a food security policy that includes expansion of agriculture on its territory^11^, currently mainly covered by boreal forests. However, appropriation of new agricultural areas toward global food security requires also a factual and realistic discourse about food security demand as the main trigger for land conversion and global biodiversity loss^54^, contextualized by rates of increase of world population^55,56^. Population growth rates are geographically strongly variable^57^ and often largest in regions that are predicted to experience negative changes in agricultural productivity^1–3^. Given our findings, intensified research should be conducted about the requirements for agronomic activities in areas where enhanced GDD_5_ ≥ 1200 conditions are expected. Such research will need to be interdisciplinary, integrating climate science with plant, soil, water and ecological sciences along with socio-economic research in order to recognize opportunities, address the challenges, and minimize and manage undesirable consequences of agriculture at higher latitudes.

## Methodology

We employed seven GCMs to quantify the impact of temperature changes on the shift in GDD_5_ across the global boreal region: cccma_cgcm31 (Canada), csiro_mk30 (Australia), ipsl_cm4 (France), mpi_echam5 (Germany), ncar_ccsm30 (USA), ukmo_hadcm3 (United Kingdom), and ukmo_hadgem1 (United Kingdom) (Extended Data)^27^ using ClimGen^58^. Input data series are available at https://crudata.uea.ac.uk/~timo/climgen/data/questgsi/. *ClimGen* allows a geographical allocation of projected isotherms, enabling the calculation of spatial and temporal variability of GDD_5_ and its scenario-induced uncertainties (see Extended Data). Monthly temperature projections were generated for the period of 2040 to 2099 using 0.5 × 0.5 degree grid resolution, which is the highest resolution for contemporary GCMs. They were interpolated into a global temperature high-resolution raster surface dataset, using the Natural Neighbour interpolation method (ArcGIS v. 10.4 Spatial Analyst toolset). To attenuate annual variations, the monthly values of five consecutive years were separately averaged (e.g. January 2040–2044). Since no daily resolution was given, the months with average values >5 °C were used to calculate daily GDD_5_ averages using days-per-month multipliers. Finally, these calculated daily values of each 5-year period were summarized to obtain annual GDD_5_ values for agricultural projections^9,16,17,19–21^. No upper temperature boundary was set for GDD_5_ ≥ 1200. This procedure was carried out for all models using either emission based or transient GHG based scenarios. Results from the emission based and transient GHG scenarios were statistically similar (Fisher test and Student-t test, see Extended Data). Climate projections^59^ carried out for seven Global Climate Models (GCMs), for both emission GHGs and transient GHGs were employed to calculate the global distribution of GDD values relevant to the growth of small cereals (GDD_5_ ≥ 1200). Each of the seven models was given equal weighting (1/N)^30^ for all analyses within our model-averaging approach. While the transient CO_2_-based scenarios are based on the assumption of constant changes over 20 years^60^ with lower shorter-term uncertainties in the climate parameters^61^, the emission-based scenarios take future socio-economic conditions into account, such as population growth and technological developments to estimate emissions of GHGs resulting from human activities^62^. A statistical test carried out for the 12-period scenarios for both CO_2_ emission-based and transient CO_2_-based approaches showed that the two modelling approaches did not differ significantly (Extended Data, Table 2). In the case of our data this is likely to be due to the averaging of the results over 5-year periods. For simplicity of presentation, our main graphics and tables are based only on emission scenarios.

Precipitation assessments were also carried out with datasets available at QuestGSI^27^ (*ClimGen*^58^ derived) with precipitation values in the same landscape-wide gridded model setting basis that we had temperature values. Monthly mean precipitation values, also at 0.5 by 0.5° resolution, were interpolated into monthly projection rasters for further usage analogous to the GDD_5_ values. The PET was calculated using the Thornthwaite approach^63^ as previously described^64^ using locally approximated day lengths, monthly and annual temperatures. The climatic water surplus and deficit values were estimated by subtracting the PET from the corresponding precipitation. A seasonal aridification index, for the May through October periods, was calculated as the ratio between precipitation and PET (p/PET)^23^.

More details on methodology and intermediary steps can be found in the Extended Data. All maps were created using ESRI’s ArcGIS (ArcMap) version 10.4 Desktop (http://www.esri.com)^65^. The two boreal region layers were obtained from Brandt^66^ and Potapov *et al*.^67^.

## Electronic supplementary material

Extended data
