# Supplementary material for: Northward shift of the agricultural climate zone under 21st-century global climate change
Source: Sci Rep. 2018 May 21;8:7904. doi: 10.1038/s41598-018-26321-8 (PMC5962595; doi:10.1038/s41598-018-26321-8)
Supplement: Supplementary file 1 — Extended data [file 41598_2018_26321_MOESM1_ESM.docx]

**Northward shift of the agricultural climate zone under 21^st^-century global climate change**

Myron King^1^, Daniel Altdorff^2^, Pengfei Li^3,4^, Lakshman Galagedara^2^, Joseph Holden^4^, Adrian Unc^2^

**Affiliations**

^1^Environmental Policy Institute, School of Science and the Environment, Memorial University of Newfoundland, Corner Brook NL, A2H 4G5, Canada

^2^School of Science and the Environment, Memorial University of Newfoundland, Corner Brook NL, A2H 4G5, Canada

^3^College of Geomatics, Xi’an University of Science and Technology, Xi’an 710054, China

^4^water@leeds, School of Geography, University of Leeds, Leeds, LS2 9JT, UK

**EXTENDED DATA**

**METHODOLOGY DETAILS**

*GDD calculations*

We used climate projections produced by seven global climate models (GCMs) (Extended Data, Table 1).

Extended Data, Table 1. Summary of the GCMs employed; input datasets for the models are available at https://crudata.uea.ac.uk/~timo/climgen/data/questgsi/.

| Coupled Model Intercomparison Project, phase 3 GCM (CMIP3 GCM)^S1^ | | ClimGen Scenarios (years) | Reference |
| --- | --- | --- | --- |
| Model ID | Agency / Model (country) |  |  |
| cccma_cgcm31 | The Third Generation Coupled Global Climate Model,  Canadian Centre for Climate Modelling and Analysis, version 3.1 (Canada) (https://www.canada.ca/en/environment-climate-change/services/climate-change/centre-modelling-analysis/models/third-generation-coupled-global.html) | CO_2_ emissions based  (2040-2099),  Transient CO_2_ based  (2040-2099),  Timeslice  (2040-2069) | Quest-GSI^27^ |
| csiro_mk30 | Commonwealth Scientific and Industrial Research Organisation (CSIRO) Atmospheric Research, Mk3 model (Australia)^29^ |  |  |
| ipsl_cm4 | Institut Pierre Simon Laplace Climate Modelling Centre, Climate Model 4 (France)^28^ (cmc.ipsl.fr/ipsl-climate-models/) |  |  |
| Mpi_echam5 | Max Planck Institute for Meteorology, atmospheric general circulation model ECHAM, version 5 (Germany)  (www.mpimet.mpg.de/en/science/models/mpi-esm/echam/) |  |  |
| ncar_ccsm30 | National Center for Atmospheric Research, Community Climate System Model, version 3.0 (USA) (www.cesm.ucar.edu/models/ccsm3.0/) |  |  |
| ukmo_hadcm3 | United Kingdom Met Office, Hadley Centre Coupled Model, version 3 (United Kingdom) **(**www.metoffice.gov.uk/research/modelling-systems/unified-model/climate-models/hadcm3**)** |  |  |
| ukmo_hadgem1 | United Kingdom Met Office, Hadley Centre Global Environmental Model, version 1 (United Kingdom) (www.metoffice.gov.uk/research/modelling-systems/unified-model/climate-models/hadgem1) |  |  |

In this analysis, we considered global observed temperature from 1901 to 2005 as the baseline input parameter. The climate projections for the seven employed GCMs were generated by *ClimGen*^58^. They were further processed under the NERC QUEST-GSI (Global-Scale Impacts)^27^ project (2007-2011), as made available online (http://www.met.reading.ac.uk/research/quest-gsi). *ClimGen* takes observational input data (e.g. temperature, precipitation, vapour pressure, wet-day frequency)^59^, through model processing, creates global point grid data at 0.5˚ x 0.5˚ resolution for multiple GCM-related variables including temperature, precipitation, vapour pressure, and wet-day frequency among others.

Our quantitative GCM data analysis results were obtained as follows:

- Global average temperature raster data files (61 MB, 32-bit, single band, high resolution 6443 x 2499 global point grid interpolation per file) were created for each month of the year from 2040 to 2099 for each GCM scenario (emissions-based transient and prescribed-change transient, a total of 14 scenarios; Extended Data Table 1), and for each month of the year from 2040 to 2069 for a third scenario (prescribed-change timeslice). Globally expansive minimum temperature raster data files were completed in the same manner utilizing the same interpolation methods. All adopted scenario output grid files were processed with a prescribed global temperature change of +2 °C by 2050 as defined originally through the NERC QUEST-GSI project^27^.
- Natural neighbour interpolation average temperature rasters contributed to the further development of 5-year period average GDD_5_ rasters for analysis related to agriculture potentials as noted. GDD_5_ raster data files with the same properties as the above global average temperature rasters were created. Initially twelve GDD_5_ rasters were created for each GCM model of the emissions-based transient and prescribed-change transient scenarios. Six GDD_5_ rasters were created for each GCM model of the third prescribed-change timeslice scenario.
- As a basis for period comparison, observed temperatures that were used in the ClimGen climate scenario generator to produce the various GCM model outcomes were also processed. The 5-year temperature period from 2001-2005 was used to create the current GDD_5_ picture (observed data) using the same interpolation and GDD_5_ calculation treatment as the CC scenarios.


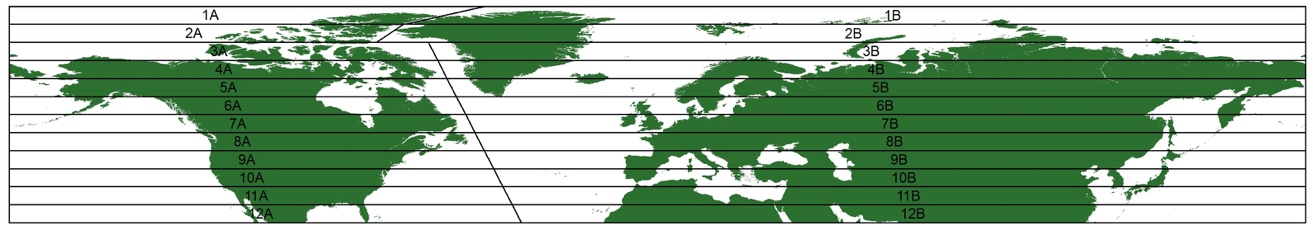


Extended Data, Figure 1. The 5˚ latitude grid employed in estimating northward shift of GDD_5_ ≥1200 regions. E.g. slice 8 describes the 45-50° N region. Note the separate treatment of North America and Eurasia. Map created using ArcGIS Desktop v. 10.4.1^65^.

With land surface areas divided into latitudinal parcels, we carried out the following steps:

- Calculated yearly average GDD_5_ over 5 year periods (12 periods available for CO_2_ emissions and transient CO_2_ scenarios, 6 available for the timeslice scenario)
- Filtered GDD_5_ to lower limits to match known crop-specific values (e.g. GDD_5_ ≥1200).

**Latitudinal approach:**

- Calculated the total geographical area (in km^2^) for each latitudinal parcel corresponding to the filtered 5-year average GDD_5_ rasters for each GCM of each 12-period scenario.
- Calculated average, maximum and minimum parcel area as predicted by all GCMs, for each period.
- Extracted areal values for change comparison with the baseline period (2001-2005).

This processing allowed us to analyse the changing amount of GDD_5_ area per latitude slice with changing time period.

**Country approach:**

A second land surface division approach was also undertaken, based on a boreal forest ecoregion representation, adapted after Potapov et al., 2008^67^ and Natural Resource Canada (http://www.nrcan.gc.ca/forests/boreal/14252)^66^. This approach used the same steps as the above, but then employed a country areal approach to the sub-areal processing:

- Calculated the total geographical area (in km^2^) for each country (or part-country area) which also contained boreal ecoregion area, corresponding to the filtered 5-year average GDD_5_ rasters for each GCM of each 12-period scenario.
- Calculated average, maximum and minimum parcel area as predicted by all GCMs, for each period.
- Extracted areal values for change comparison with the baseline period.

For both longitudinal and country approaches, we worked with the 12-period scenarios only, which provided a greater number of periods, with predictions reaching further into the future. For final results and discussion, emphasis was placed on the 12-period CO_2_ emissions scenarios. While the transient CO_2_-based scenarios balance assumptions of constant changes over 20 years with shorter term uncertainties in the climate parameters^61^, a statistical test carried out against the 12-period scenarios for both CO_2_ emission-based and transient CO_2_-based approaches has shown that they did not differ significantly (Extended Data, Table 2). In the case of our data this is likely due to the fact that we averaged our results over 5-year periods.

All maps were created using ArcGIS Desktop version 10.4.1^65^. Unless differently mentioned, all maps were obtained based on the non-weighted average of the outputs from all GCM’s.

Extended Data, Table 2: The 12-period CO_2_ emissions scenario and the 12-period transient scenario results were checked using a statistical F test and Student’s t test (three different time periods are shown). GCM model ranges for emissions time periods and transient time periods were first shown to have equal variance (row 1, F test), and then shown to have no statistically significant difference in means (row 2, t test).

|  |  |  | Period | | | | | |  |
| --- | --- | --- | --- | --- | --- | --- | --- | --- | --- |
|  |  |  | 2050 - 2054 | | 2070-2074 | | 2095-2099 | |  |
|  | |  | *Emissions* | *Transient* | *Emissions* | *Transient* | *Emissions* | *Transient* |  |
|  |  |  | **F-test: Variances are equal if F < F(Critical one-tail)** | | | | | |  |
|  |  | Mean | 8903375 | 8982187 | 10093373 | 10247310 | 11011531 | 11097609 |  |
|  |  | Variance | 1.29×10^12^ | 6.22×10^11^ | 1.70×10^12^ | 7.89×10^11^ | 2.21×10^12^ | 8.23×10^11^ |  |
|  |  | Observations | 7 | 7 | 7 | 7 | 7 | 7 |  |
|  |  | df | 6 | 6 | 6 | 6 | 6 | 6 |  |
|  |  | F | 2.08 |  | 2.16 |  | 2.69 |  |  |
|  |  | P(F<=f) one-tail | 0.20 |  | 0.19 |  | 0.13 |  |  |
|  |  | F Critical one-tail | 4.28 |  | 4.28 |  | 4.28 |  |  |
|  | |  | | TRUE |  | TRUE |  | TRUE |  |
|  | | **Student’s t-test (assuming equal variances). Means are equal if –t (Critical two tail) < t Stat < +t (Critical two tail)** | | | | | | |  |
|  | | Mean | 8903375 | 8982187 | 10093373 | 10247310 | 11011531 | 11097609 |  |
|  |  | Variance | 1.29×10^12^ | 6.22×10^11^ | 1.70×10^12^ | 7.89×10^11^ | 2.21×10^12^ | 8.23×10^11^ |  |
|  |  | Observations | 7 | 7 | 7 | 7 | 7 | 7 |  |
|  |  | Pooled Variance | 9.56×10^11^ |  | 1.25×10^12^ |  | 1.52×10^12^ |  |  |
|  |  | df | 12 |  | 12 |  | 12 |  |  |
|  |  | t Stat | -0.15 |  | -0.26 |  | -0.14 |  |  |
|  |  | P(T<=t) one-tail | 0.44 |  | 0.40 |  | 0.45 |  |  |
|  |  | t Critical one-tail | 1.78 |  | 1.78 |  | 1.78 |  |  |
|  |  | P(T<=t) two-tail | 0.88 |  | 0.80 |  | 0.90 |  |  |
|  |  | t Critical two-tail | 2.179 |  | 2.179 |  | 2.179 |  |  |
|  | | | | TRUE |  | TRUE |  | TRUE |  |


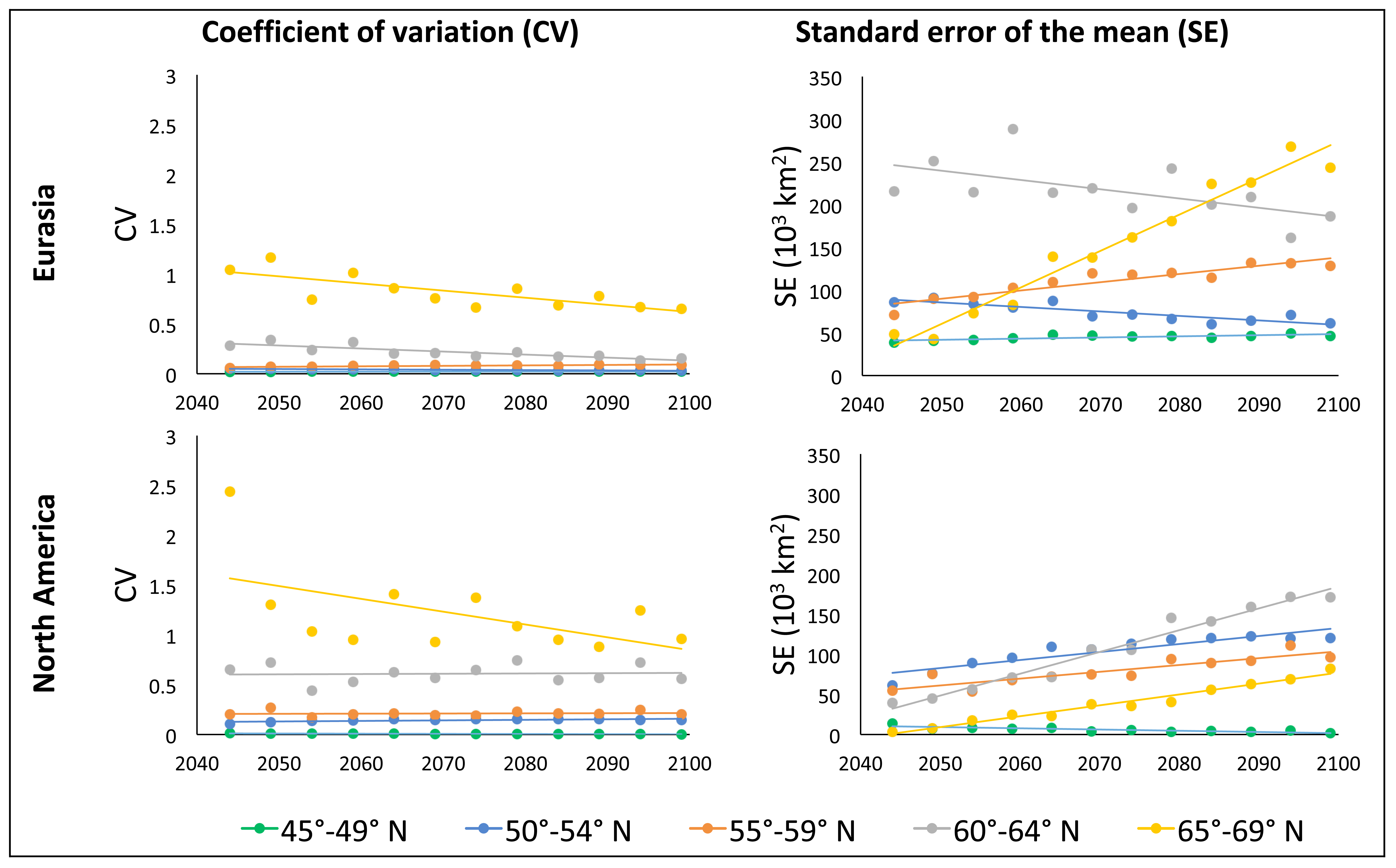


Extended Data Figure 2. Inter-model variability. Calculations were carried out for the GDD_5_ ≥1200 area projections obtained for emissions GHG scenarios the seven GCMs.


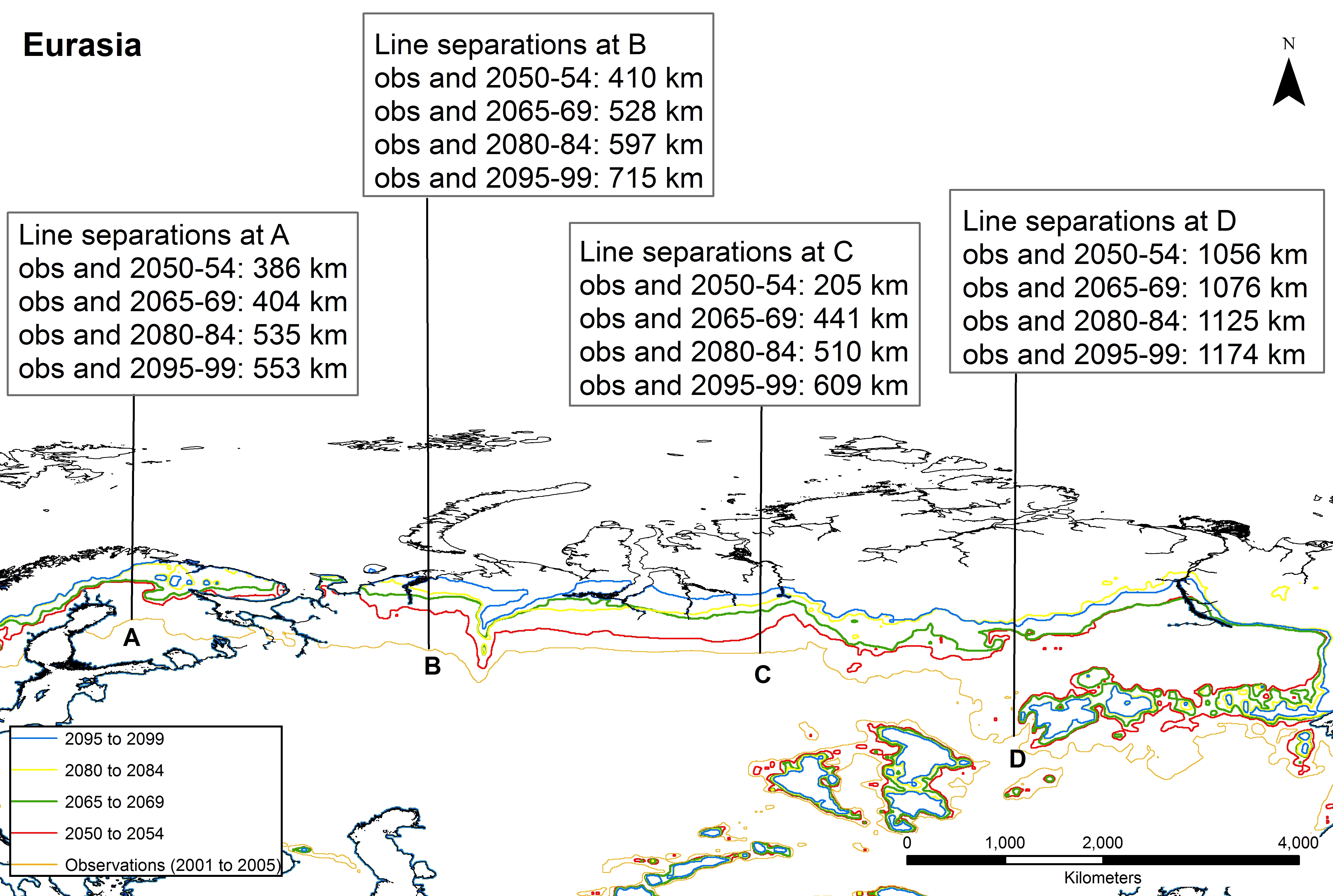


Extended Data, Figure 3. Examples of the variability in the linear northward shift across selected regions. Average GDD_5_ ≥1200 boundaries. Map created using ArcGIS Desktop v. 10.4.1^65^.


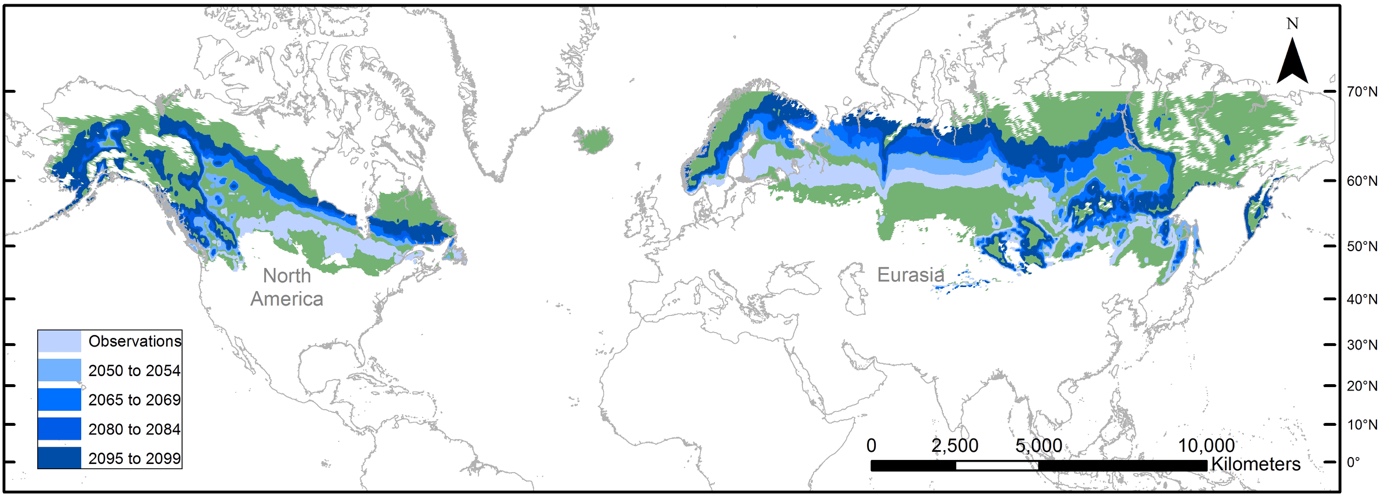


Extended Data, Figure 4. Description of observations (2001-2005) and projected GDD_5_ for the selected periods. While we are primarily interested in GDD_5_ ≥1200, we here employ an additional upper GDD_5_ threshold value of 1400 to aid in period comparison, with some overlap between periods still evident under threshold. The boreal forest ecoregion (green background) was adapted after Potapov et al., 2008^67^ and Natural Resource Canada (http://www.nrcan.gc.ca/forests/boreal/14252)^66^, with data for the latter accessed under the Open Government Licence-Canada (https://open.canada.ca/en/open-government-licence-canada). Map created using ArcGIS Desktop v. 10.4.1^65^.

*Precipitation estimates*

All precipitation data were acquired initially in a 0.5 x 0.5-degree global grid format, as previously done for the temperature data. The source was QuestGSI^27^ (Climgen^58^ derived). Monthly mean precipitation values were interpolated into monthly rasters for further use.

*Potential Evapotranspiration (PET):*

The PET was calculated using the Thornthwaite method^63^ suitable for boreal systems^64^. Briefly, we first used previously gridded and interpolated mean monthly temperature data from QuestGSI^27^ (Climgen^5^ derived) to calculate a Monthly Thornthwaite Heat Index (i); the Annual Heat Index was then found by summing the Monthly Heat Indices for each year; in parallel, an uncorrected PET estimation for each month was calculated (based on an assumed 30-day month with 12 hours of daylight per day; this uncorrected PET was calculated using the mean temperature for the month in question along with the Annual Heat Index previously found). Finally, we applied a correction to PET (uncorrected) using the real number of days per month and a monthly averaged value of day length for 5-degree latitudinal intervals between 40 and 70 degrees. (Using a 5 degree latitude resolution caused the artificial stripes in the PET and surplus/deficit maps).

All precipitation and PET calculation steps were carried out within the GIS tooling system, applied to each smallest unit for each temperature raster as needed. Precipitation and PET rasters were clipped with our previously used Worldwide Boreal Zone^66,67^ interest area, for incorporation into our final mapping products. **EXTENDED RESULTS**


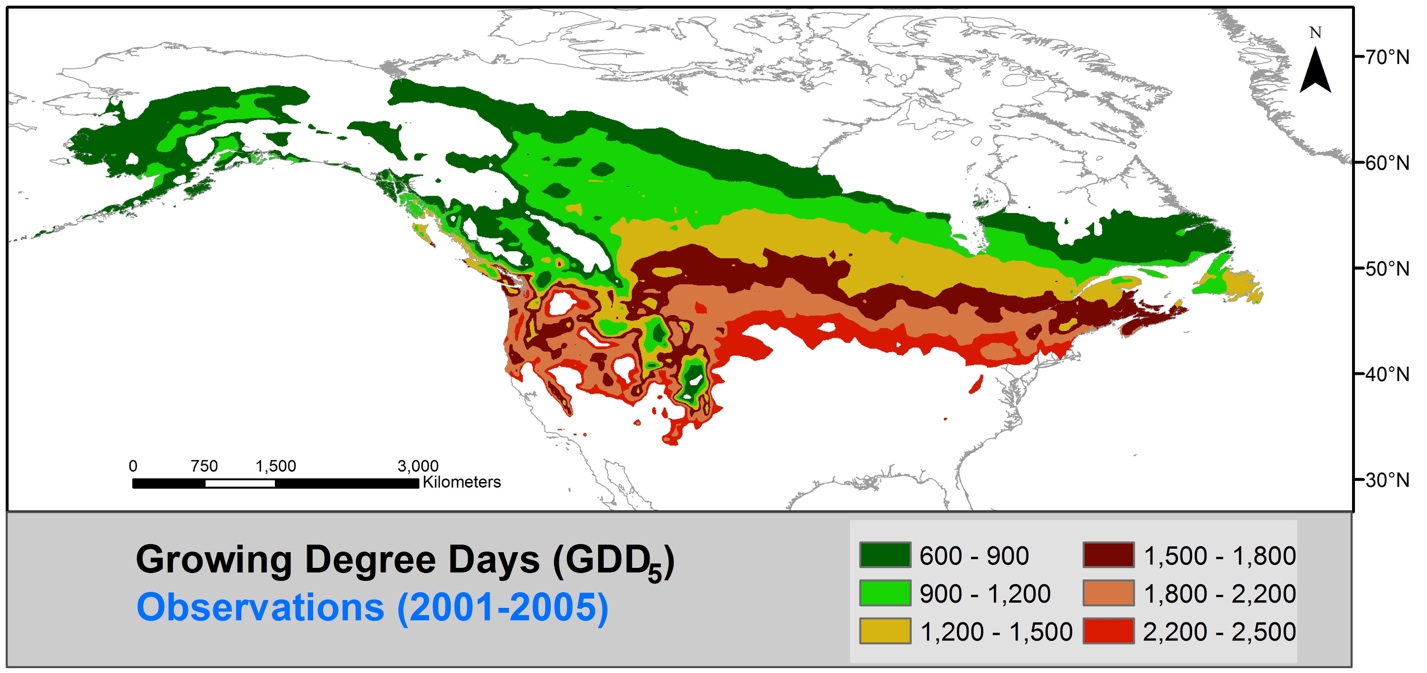


Extended Data, Figure 5. Observed GDD_5_ distribution for the boreal region on North America; average for 2001-2005 period. Map created using ArcGIS Desktop v. 10.4.1^65^.


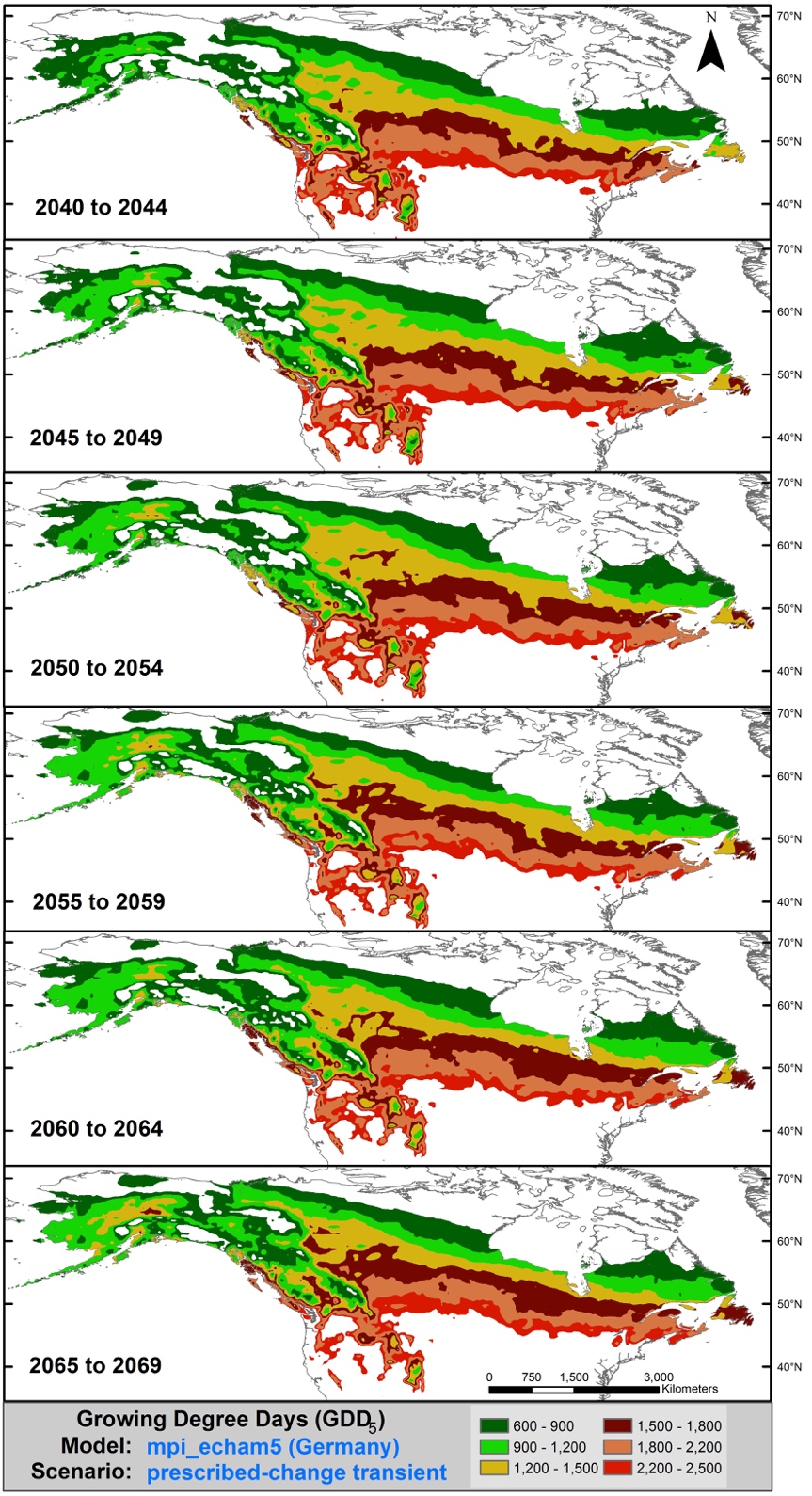

Extended Data, Figure 6. Forecasted GDD_5_ distribution for the North America boreal region. Results were obtained for seven GCMs using three different model scenarios. GCM mpi_echam5 ‘Germany’ under the prescribed-change transient scenario is shown. Map created using ArcGIS Desktop v. 10.4.1^65^.


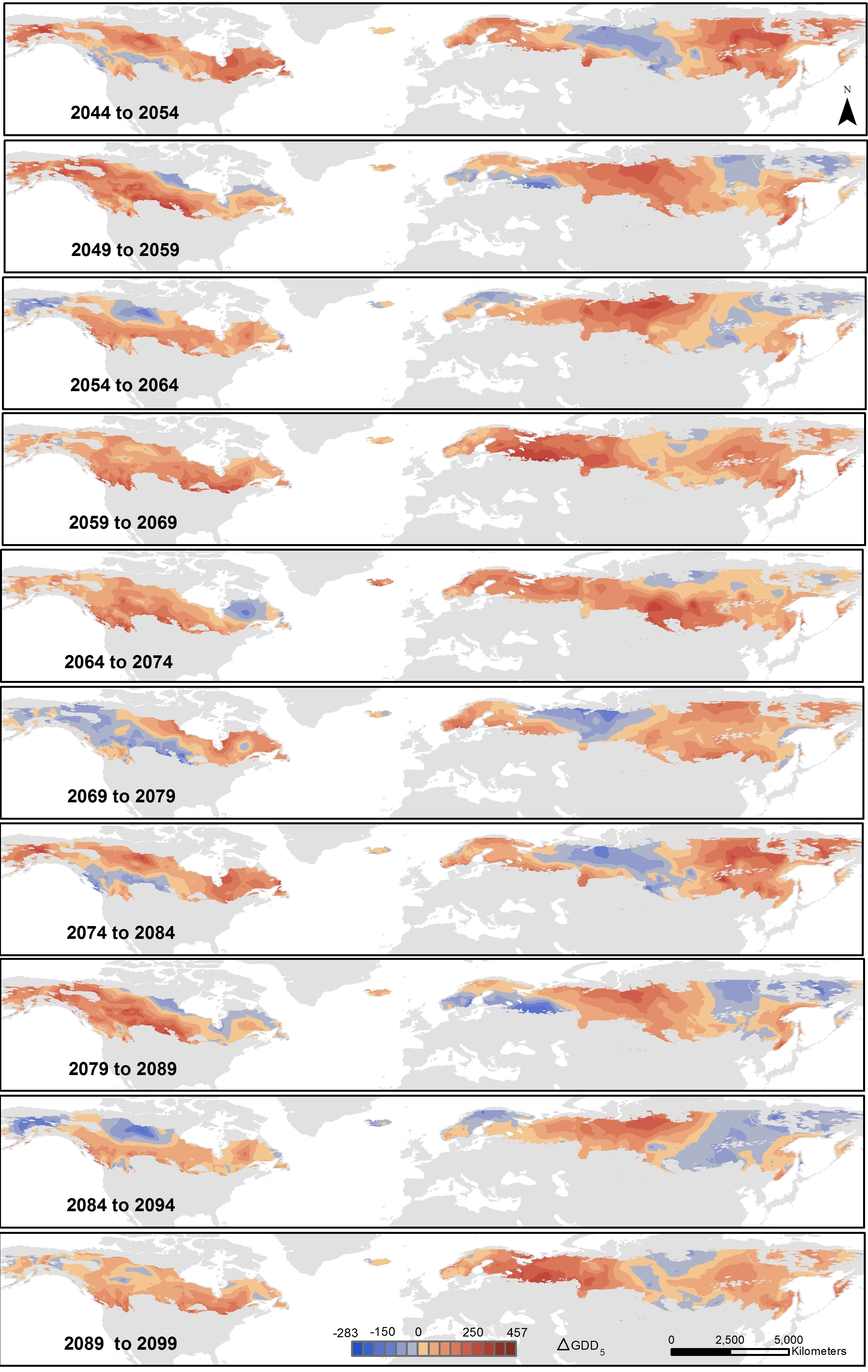


Extended Data, Figure 7. Spatial variability of GDD_5_ ≥1200 changes within the boreal region^66,67^ for selected 10-year periods. Each map displays the difference between two 5-year period average values (e.g. 2049 stands for the period 2045-49). Values were obtained by averaging the emission CO_2_-based predictions for the seven GCMs. Map created using ArcGIS Desktop v. 10.4.1^65^.


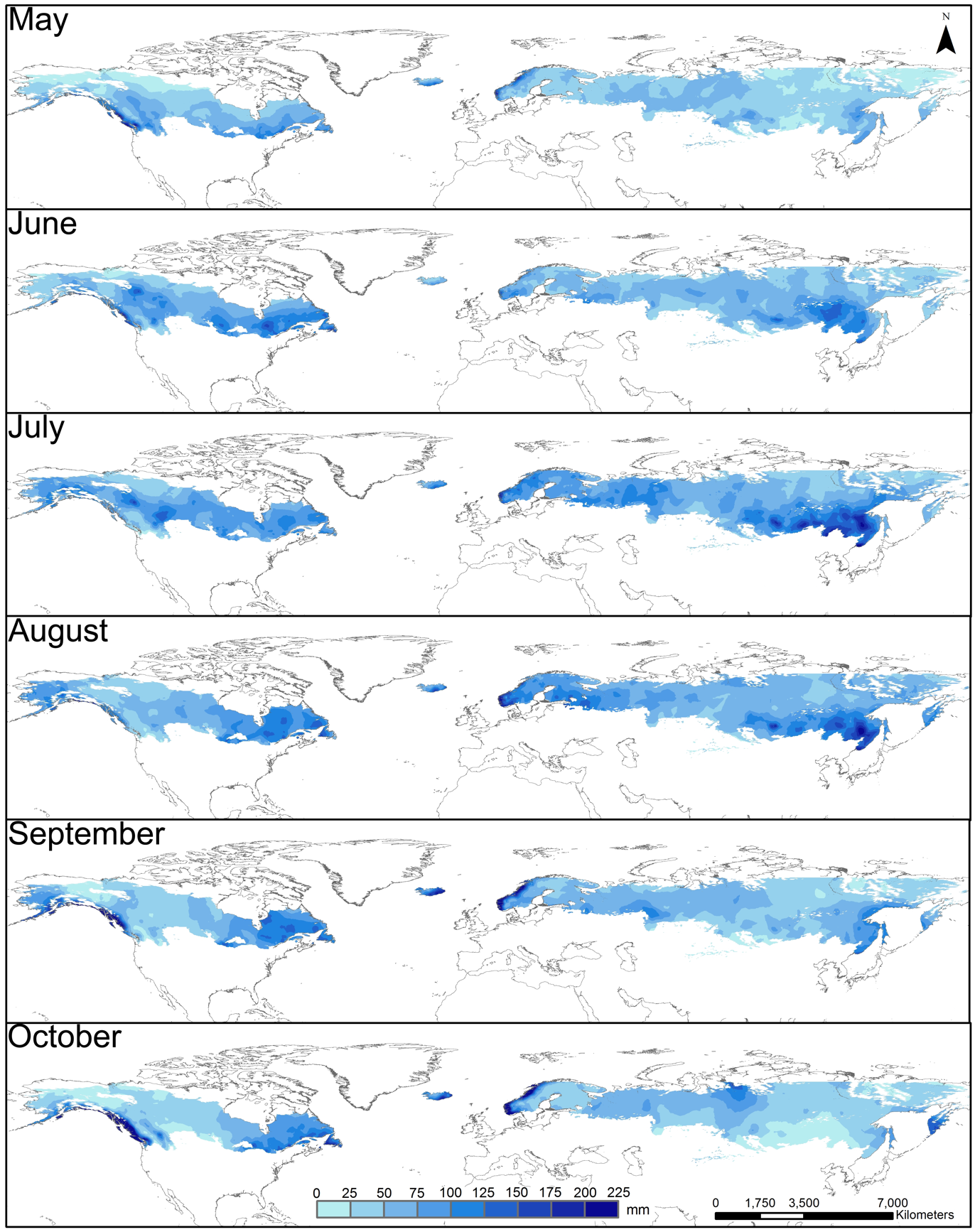


Extended Data, Figure 8. Projected monthly precipitation for the 2095-2099 period. Map created using ArcGIS Desktop v. 10.4.1^65^.


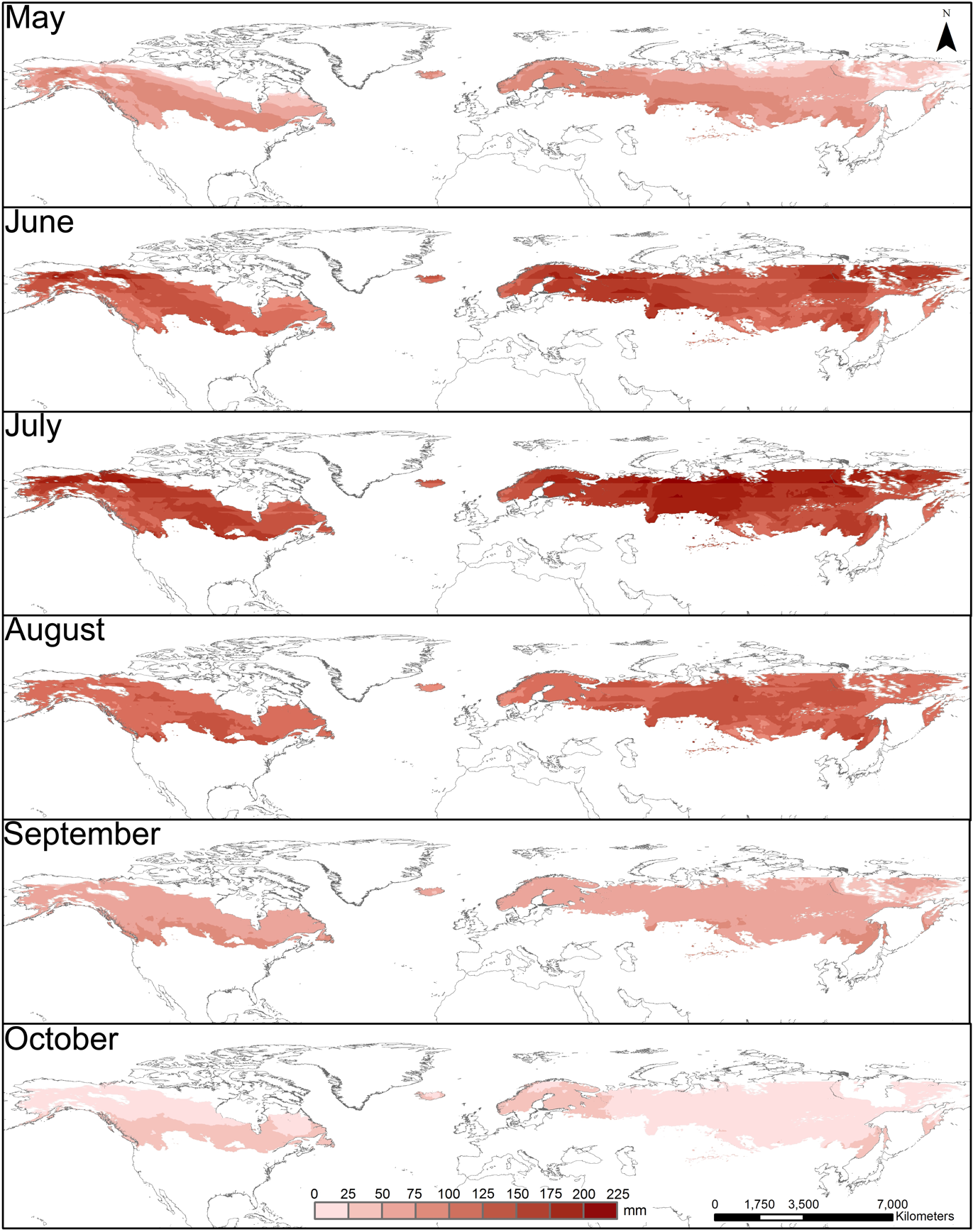


Extended Data, Figure 9. Projected potential evapotranspiration for the 2095-2099 period. Map created using ArcGIS Desktop v. 10.4.1^65^.


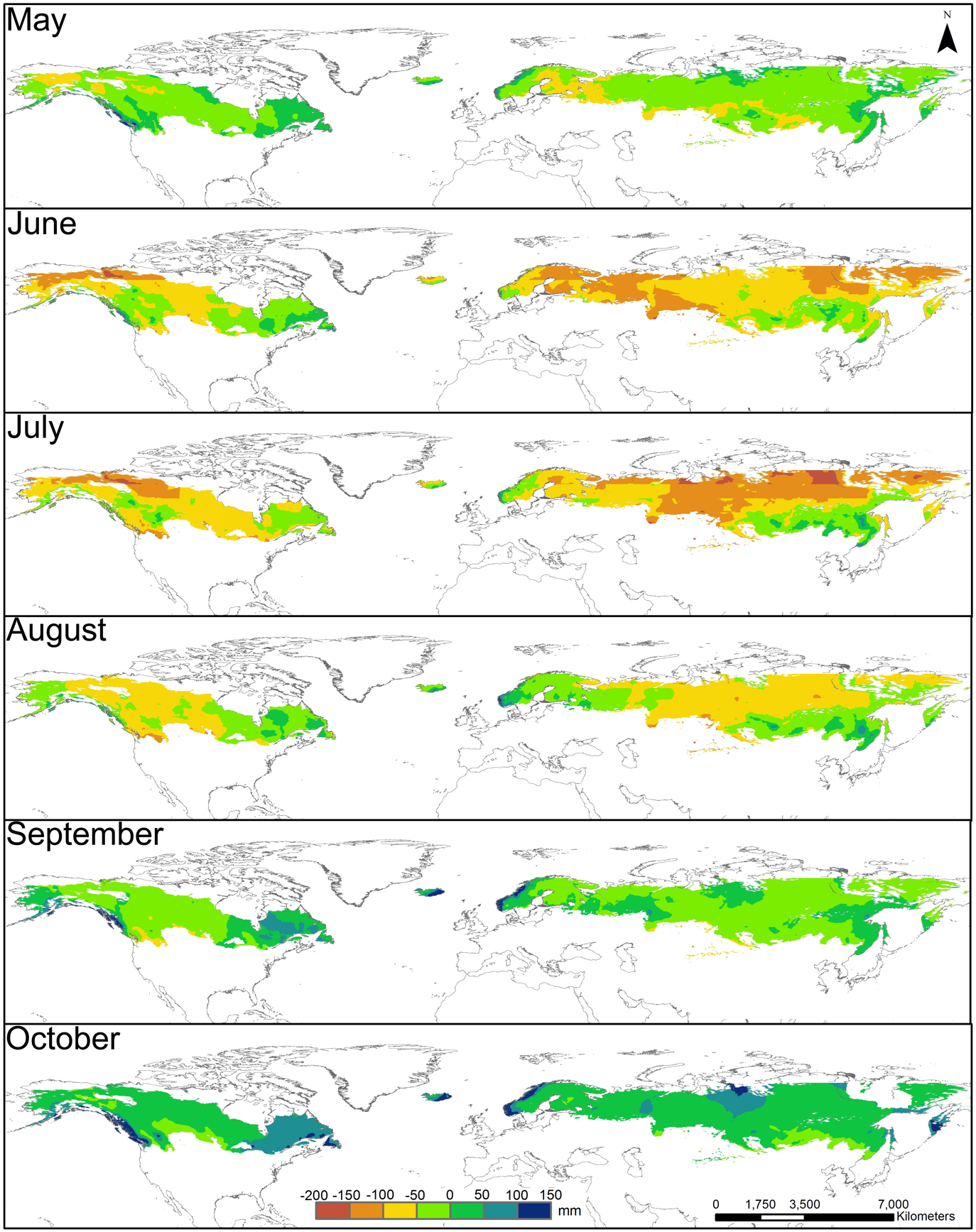


Extended Data, Figure 10. Projected monthly water balance for the 2095-2099 period. This has been calculated by deducting PET from precipitation (Extended Data, Figures 8 and 7, respectively). Map created using ArcGIS Desktop v. 10.4.1^65^.


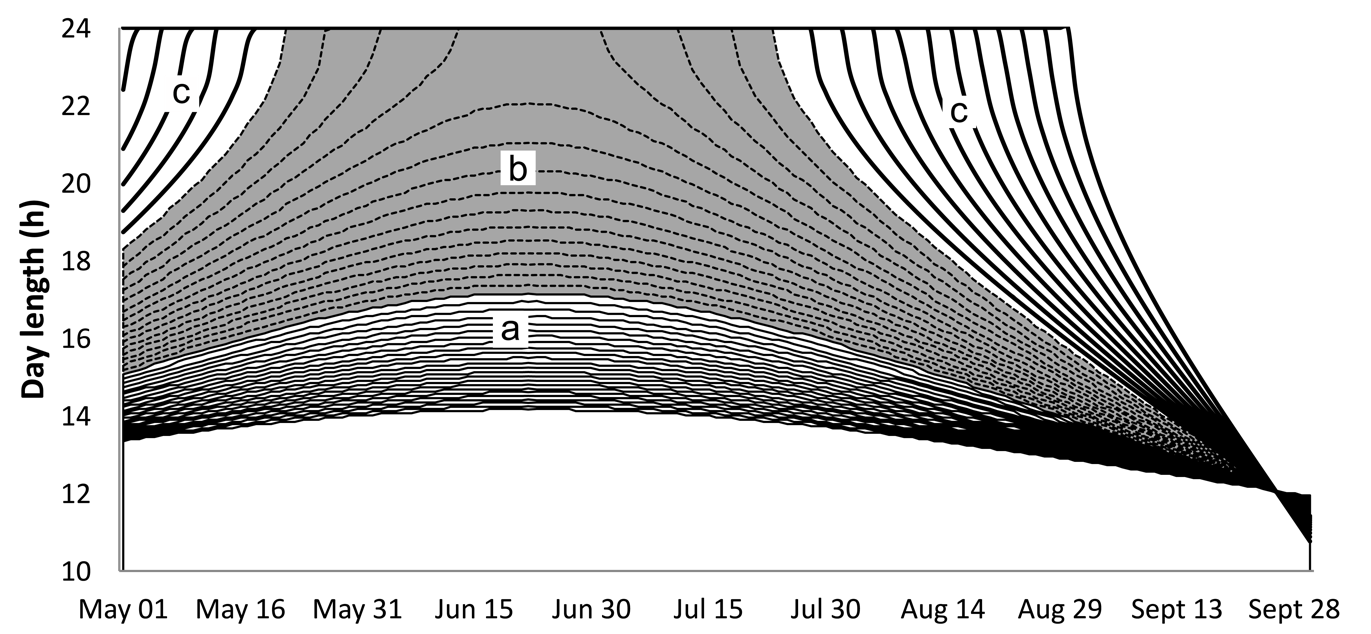


Extended Data, Figure 11. Photoperiod distribution for the May 1^st^ to September 30^th^ period across latitudes. Day lengths were calculated with the R software^S2^ using the geosphere package^S3^; a, covers the region from of 30 to 54° N, b, covers the region from 55 to 69° N; c, covers the region from 70 and 85° N.

Extended Data, Table 3. Water balances as the difference between average precipitation and average PET. National areas as observed (2001-2005) and projected (2095-2099); values are based on the projected land area with GDD_5_ ≥1200. Projected data reflects the average obtained from the seven GCMs. The ten countries with the largest boreal area are listed here.

| **Country** | **Boreal**  **area**  **(km2)** | **Water Balance (Precipitation – PET) (mm)** | | | | | | | | | | | | |
| --- | --- | --- | --- | --- | --- | --- | --- | --- | --- | --- | --- | --- | --- | --- |
|  |  | **x ≤ (-300)** | | **(-300) ≤ x < (-100)** | | | **(-100) ≤ x < (100)** | | | **(100) ≤ x < (300)** | | | **x ≥ (300)** | |
|  |  | Obs. | 2095-99 | Obs. | 2095-99 | | Obs. | 2095-99 | | Obs. | 2095-99 | | Obs. | 2095-99 |
|  |  | **Area with GDD_5_ ≥1200 (km^2^)** | | | | | | | | | | | | |
| Russian Federation | 11,526,702 | 81,028 | 5,633,824 | 3,114,919 | 2,857,782 | | 642,535 | 472,932 | | 19,787 | 45,596 | | 0 | 0 |
| Canada | 6,532,377 | 0 | 1,873,722 | 348,629 | 1,751,869 | | 1,006,226 | 994,735 | | 282,191 | 90,856 | | 14,190 | 35,362 |
| United States | 1,243,580 | 17,735 | 349,010 | 88,376 | 400,297 | | 185,460 | 49,197 | | 5,129 | 21,911 | | 495 | 26,824 |
| Mongolia | 556,492 | 22,024 | 156,558 | 133,339 | 227,974 | | 4,772 | 2,896 | | 0 | 0 | | 0 | 0 |
| China | 486,061 | 17,009 | 51,569 | 155,924 | 388,257 | | 152,533 | 18,642 | | 0 | 0 | | 0 | 0 |
| Finland | 329,204 | 0 | 307,418 | 164,002 | 1,549 | | 0 | 0 | | 0 | 0 | | 0 | 0 |
| Sweden | 309,929 | 0 | 69,047 | 17,647 | 128,185 | | 4,517 | 0 | | 0 | 0 | | 0 | 0 |
| Norway | 279,859 | 0 | 2,689 | 4,215 | 31,491 | | 9,789 | 31,609 | | 1,995 | 16,141 | | 4,900 | 25,661 |
| Kazakhstan | 124,648 | 31,835 | 96,129 | 56,391 | 11,417 | | 0 | 0 | | 0 | 0 | | 0 | 0 |
| Iceland | 102,382 | 0 | 0 | 0 | 0 | | 0 | 0 | | 0 | 0 | | 0 | 0 |
| Kyrgyzstan | 18,241 | 188 | 15,731 | 4,738 | 0 | | 0 | 0 | | 0 | 0 | | 0 | 0 |
| **Summary areas (km^2^)** | | | | | | | | | | | | | | |
|  | | | | Eurasia | | | | | North America | | | | | |
| Total Boreal Area | | | | 13,733,517 | | | | | 7,775,957 | | | | | |
| Boreal area  with GDD_5_ ≥1200 |  | | | Observed | | 2095-99 | | | Observed | | | 2095-99 | | |
|  | Total | | | 4,644,089  (33.8%) ^(1)^ | | 10,593,097 (77.1%) | | | 1,948,431 (25.1%) | | | 5,593,782 (71.9%) | | |
|  | Seasonal water surplus  (>100mm) ^(2)^ | | | 26,683  (0.6%) ^(3)^ | | 87,398  (0.8%) | | | 302,005  (15.5%) | | | 174,953  (3.1%) | | |
|  | Seasonal balanced water budget  (-100mm to +100mm) | | | 814,147  (17.5%) | | 526,079  (5%) | | | 1,191,686 (61.2%) | | | 1,043,932 (18.7%) | | |
|  | Seasonal water deficiency (<100mm) | | | 3,803,258  (81.9%) | | 9,979,619  (94.2%) | | | 454,740  (23.3%) | | | 4,374,898 (78.2%) | | |
| ^(1)^ Boreal area with GDD_5_ ≥1200 as percentages of total boreal area  ^(2)^ Water balance calculated as Precipitation-PET  ^(3)^ Percentages of total boreal area with GDD_5_ ≥1200  NOTE: due to round-up errors the values do not necessarily add to 100% | | | | | | | | | | | | | | |

Extended Data, Table 4. Seasonal aridification index (precipitation divided by PET, p/PET, for the period of May through October). The median and median absolute deviation (MAD) of the aridification index for the entirety of national areas, both as observed (2001-2005) and projected (2095-2099). Values of <0.65 are considered to be associated with aridification^23^.

| Country | Aridification index (p/PET; Median ± MAD) | |
| --- | --- | --- |
|  | Observed (2001-2005) | Projected (2095-2099) |
| Russian Federation | 0.68 ± 0.09 | 0.52 ± 0.11 |
| Canada | 1.04 ± 0.18 | 0.59 ± 0.19 |
| United States | 1.00 ± 0.23 | 0.57 ± 0.20 |
| Mongolia | 0.48 ± 0.10 | 0.53 ± 0.11 |
| China | 0.79 ± 011 | 0.72 ± 0.09 |
| Finland | 0.65 ± 0.02 | 0.49 ± 0.04 |
| Sweden | 0.77 ± 0.04 | 0.60 ± 0.06 |
| Norway | 0.93 ± 0.36 | 1.02 ± 0.34 |
| Kazakhstan | 0.48 ± 0.08 | 0.34 ± 0.07 |
| Kyrgyzstan | 0.48 ± 0.03 | 0.30 ± 0.03 |


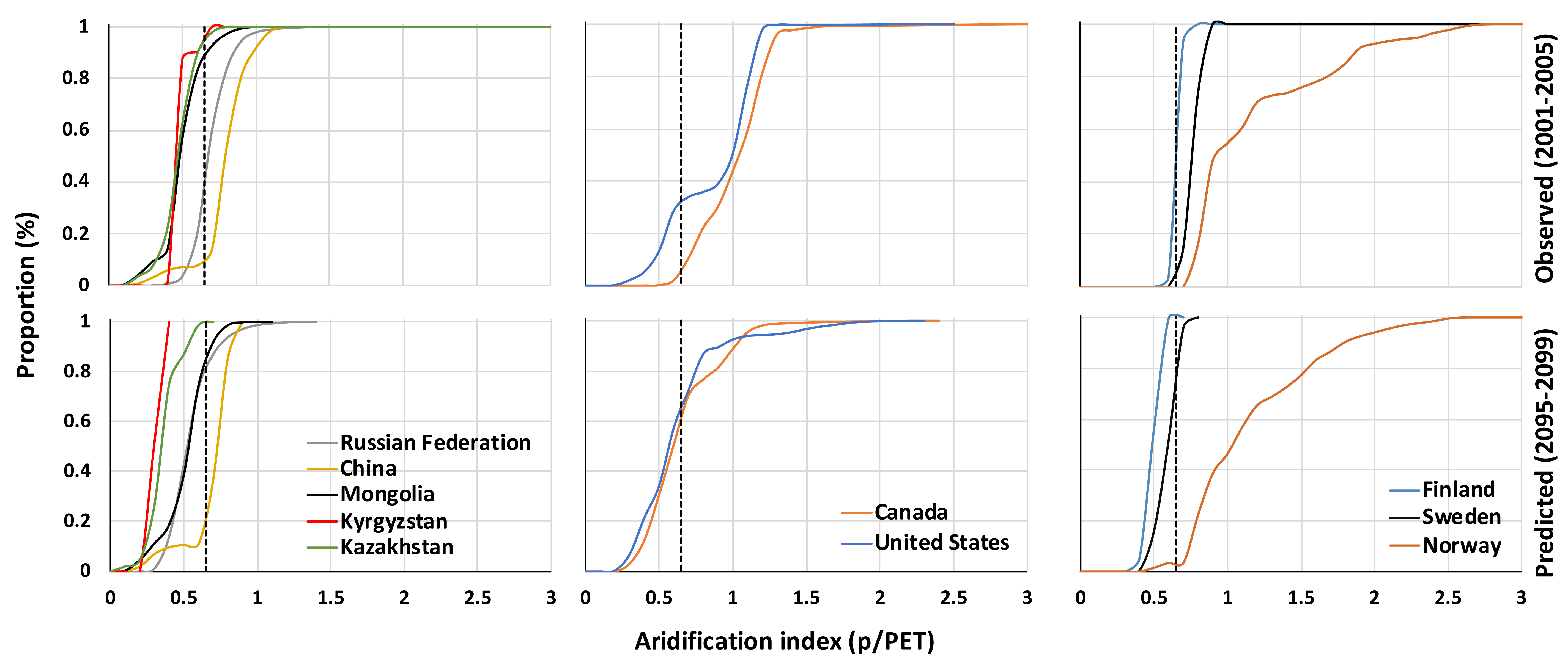


Extended Data Figure 12. Proportional regional distribution of the seasonal aridification index (p/PET) for the observed (2001-2005) and projected (2095-2099) periods. The vertical dotted lines describe the aridification threshold (p/PET=0.65)^23^.

**SUPPLEMENTARY REFERENCES:**

S1 Meehl, G. A. *et al.* THE WCRP CMIP3 Multimodel Dataset: A New Era in Climate Change Research. *Bull. Amer. Meteor. Soc.* **88**, 1383-1394 (2007).

S2 R: A language and environment for statistical computing (R Foundation for Statistical Computing, Vienna, Austria, 2017).

S3 geosphere: Spherical Trigonometry. R package version 1.5-7 (2017).
